# Treadmill training mitigates bone deterioration via inhibiting NLRP3/Caspase1/IL-1β signaling in aged rats

**DOI:** 10.1186/s12891-022-06055-5

**Published:** 2022-12-13

**Authors:** Qi Wu, Peirui Zhong, Pengyun Ning, Lu Tan, Xiarong Huang, Ting Peng, Linwei Yin, Fu Luo, Mengjian Qu, Jun Zhou

**Affiliations:** 1 Department of Rehabilitation, Hengyang Medical School, The First Affiliated Hospital, University of South China, No. 69, Chuanshan Road, Hengyang, Hunan Province 421001 Hengyang, People’s Republic of China; 2 Rehabilitation Laboratory, Hengyang Medical School, The First Affiliated Hospital, University of South China, 421001 Hengyang, Hunan China; 3 Nanjing Medical University, 211166 Nanjing, Jiangsu China; 4 Department of Rehabilitation, West China Hospital, Sichuan University, 610000 Chengdu, China

**Keywords:** Aerobic physical exercise, Osteoporosis, NLRP3-mediated inflammation, Bone microenvironment, Ageing

## Abstract

**Introduction:**

Although aerobic physical exercise may improve osteoporosis during ageing, the underlying mechanism of the favorable effects remains unclear. The aim of this study was to examine the localized and generalized proinflammatory indicators and the adaptive skeletal responses to treadmill training in aged rats to explore the potential mechanisms by which treadmill training impacts bone deterioration in a natural aged rat model.

**Materials and methods:**

A total of 24 Sprague Dawley (SD) rats were included in this study. Sixteen of all these animals were twenty-four months natural aged male SD rats, which were distributed into two groups (*n *= 8/group): AC group with sham treadmill training, and AT group with 8 weeks treadmill training. The remaining 8 were six months male SD rats matched subline and supplier, which were used as the adult control group with sham treadmill training (YC group, *n* = 8). The serum, bone marrow, fresh femur, tibia, and lumbar spine were harvested for molecular biological analysis, bone mineral density (BMD) testing, and micro-CT analysis after 8 weeks of treadmill training.

**Results:**

After 8 weeks of intervention, the results showed that treadmill training increased BMD and inhibited deterioration of bone microarchitecture of hind limb bones. Further analysis showed that treadmill training increased serum P1CP concentration and decreased serum CTX-1level. Interestingly, treadmill training down-regulated the protein expressions of proinflammatory indicators, including NLRP3, proCaspase1, cleaved Caspase1, IL-1β, and GSDMD-N, and the mRNA levels of NLRP3, Caspase1, and IL-1β of the bone marrow. In addition, treadmill training also inhibited serum TNF-α and IL-1β concentration. However, 8 weeks of treadmill training did not increase BMD and bone microarchitecture in the lumbar spine.

**Conclusion:**

Treadmill training mitigates the ageing-induced bone loss and reverses the deterioration of bone microarchitecture in hind limbs probably through inhibiting NLRP3/Caspase1/IL-1β signaling to attenuate low-grade inflammation and improve the inflammatory bone microenvironment.

**Supplementary Information:**

The online version contains supplementary material available at 10.1186/s12891-022-06055-5.

## Introduction

Osteoporosis, a global health issue, is an osteo-metabolic disease characterized by substantial loss of bone mass and microarchitecture deterioration of bone tissue, affecting bone quality and strength and increasing fracture risk [1]. It is reported that the prevalence of osteoporosis in the elders of the world was 21.7% [2], and one in 3 women and 1 in 5 men over the age of 50 years will experience an osteoporotic fracture in their lifetime [3]. Therefore, it is particularly important to explore effective intervention methods and mechanisms for senile osteoporosis. It has been recognized that during ageing, low-grade chronic inflammation (LGCI), “persistent but more subtle than the acute phase response,” contributes to accelerating biological ageing and plays a role in the initiation and progression of age-related diseases such as osteoporosis (OP) by regulating bone microenvironment [4]. The pathogenesis of osteoporosis is multifactorial. Traditionally, osteoporosis has been regarded as an estrogen deficiency-mediated disease; nevertheless, emerging data have demonstrated the significant role of systemic and local inflammation in the pathogenesis of OP. Over years, clinical studies have shown that exercise can partially prevent deterioration of bone quality and quantity in the elderly [5]. However, the precise mechanisms accounting for these favorable biological responses are unclear.

The maintenance of bone mass attributes to bone remodeling, which is tightly regulated by crosstalk between bone-forming osteoblasts and bone-resorbing osteoclasts. Osteoblastic bone formation and osteoclastic bone resorption are tightly regulated by proinflammatory cytokines in the bone microenvironment. In the elderly subjects, the elevated proinflammatory cytokines lead to a gradual loss of bone mass due to an excess of bone resorption not balanced by new bone formation [6]. Human and animal experiments have shown that bone health and ageing are negatively correlated, potentially through proinflammatory cytokines, including TNF-α and IL-1β, influencing the promotion of osteoclast activity and suppression of osteoblast activity, thus accelerating bone loss and negativity impacting bone characteristics [7]. Evidence has shown that the inflammation-associated bone loss is reversible [8]. Thus changing the inflammatory microenvironment of bone may be the direction of osteoporosis treatment. Exercise plays an important role in the intervention for osteoporosis, thus understanding the potential mechanism of physical exercise for the anti-osteoporosis was meaningful for preventing and treating osteoporosis.

Commonly, osteoporosis is defined as a skeletal condition characterized by decreased density (mass/volume) of normally mineralized bone. An alternative definition has been proposed by Harold Frost, suggesting that osteopenia is the bone’s physiological response to disuse [9]. Accordingly, exercise, in particular some new forms of it that involve high strain rates, seems to be preventing bone loss and possibly also induces increases in bone mass even at older ages. A systematic review and meta-analysis concluded that all types of exercise significantly affect bone mineral density (BMD), they also provided further evidence for the favorable effect of exercise on BMD largely independent of the type of exercise [10]. Previous studies showed that walking exercise, resistance training, muscle strengthening, aerobic exercise, and high-impact exercise all promote bone turnover, increase BMD, and prevent and manage osteoporosis [11–13]. In addition, in vivo studies showed that treadmill training could increase the BMD, trabecular bone volume as well as trabecular bone surface in the aged rats [14]. Collectively, physical exercise is effective to prevent and manage osteoporosis by increasing BMD and bone microstructure, and treadmill training is a promising exercise for the elderly to antagonize osteoporosis.

Recent evidence showed that NLRP3 inflammasome, regulating the maturation and secretion of caspase1-dependent pro-inflammatory cytokines IL-1β and IL-18, and enhancing the inflammatory response, is responsible for the chronic inflammatory microenvironment in the aged. In vivo and in vitro experiments showed that NLRP3-dependent IL-1β can accelerate osteoclastogenesis by expanding inflammatory response, and can also inhibit the expression of osteogenic-related proteins or transcription factors [15, 16]. In addition, NLRP3/Caspase1 activation in mesenchymal stem cells inhibits osteogenic differentiation [17]. Targeting NLRP3 reduced age-related experimental bone loss [15]. NLRP3/Caspase1/IL-1β signaling contributes the bone loss, and the high level of NLRP3 was also detected in aged [18]. Previous studies support the potential beneficial effects of exercise on inflammation, but the underlying mechanism remains obscure. One proposed mechanism is a reduced expression and/or activation of pro-inflammatory toll-like receptors (TLRs) on innate immune cells after exercise in an obese animal model [19]. In addition, the anti-inflammatory effects of regular exercise may be mediated via both a reduction in visceral fat mass and the induction of an anti-inflammatory environment with each bout of exercise in the chronic metabolic and cardiorespiratory diseases [20]. Recently, one study showed that exercise exerts its anti-inflammatory action by suppressing adipose tissue NLRP3 inflammasome in obese mice [21]. These reports all support that exercise might inhibit bone loss via inhibiting the NLRP3-mediated inflammatory environment. However, the effect of aerobic physical exercise on NLRP3/Caspase1/IL-1β signaling mediated inflammatory bone microenvironment and bone deterioration in the aged subjects remains unclear.

In the present study, we hypothesized treadmill training mitigates bone loss and bone microstructure deterioration by inhibiting NLRP3/Caspase1/IL-1β signaling mediated low-grade inflammation in the bone microenvironment in aged rats. To verify the hypothesis, we investigated the effects of treadmill training on morphologic, biochemical, and molecular characteristics of bone in a natural aged rat model.

## Materials and methods

### Ethics statement

Six-month-old male Sprague Dawley rats (450–550 g) and 24-month-old male rats (550–650 g) were used as an adult and aged rats, respectively. All the rats were housed in animal facilities with sufficiently controlled temperature (24 ± 1℃) and humidity (50–60%) under a 12/12-h light/ dark cycle and had access to chow and water. All procedures were strictly performed in accordance with recommendations from the Guide for the National Institutes of Health for the Care and Use of Laboratory Animals (NIH Publications No. 8023, revised 2011). All animal experiments were approved by the Ethics Committee of the First Affiliated Hospital of University of South China(reference no. 202,004,270,002) and were performed in accordance with the ethical criteria contained in the bylaws of the committee. Meanwhile, all methods reported in this study were in accordance with ARRIVE guidelines.

### Animal model

**Fig. 1:**
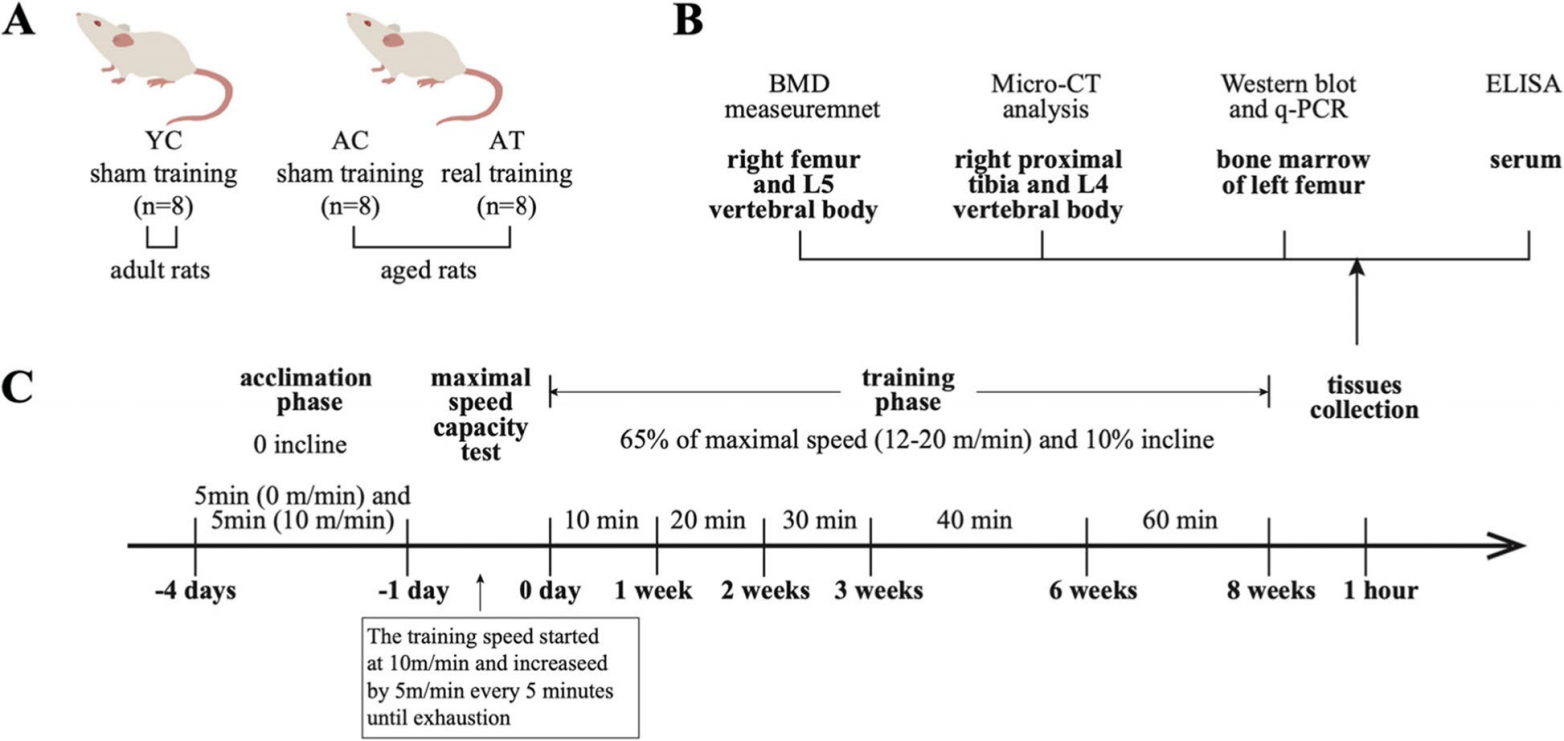
Study protocol. **A** Experimental grouping: YC, AC, and AT. The adult rats with sham training were included in the YC group (*n* = 8/group); the aged rats in the AC group and AT group received either 8 weeks of sham or real treadmill training (*n* = 8/group). **B** A schematic diagram of tissue collection and detection. **C** Experimental timeline and parameters of the training session. The rats in the AT group received real treadmill training for eight weeks, with a 3-day acclimation before training and a maximal speed capacity measurement afterward

A total of 24 Sprague Dawley rats were included in the study, including 16 aged male Sprague Dawley rats and 8 adult male Sprague Dawley rats (Chengdu DOSSY Experimental Animal Co., Ltd., Chengdu, China). The aged rats, 24-month old, were equally divided into 2 groups: natural aged model group (AC group, *n* = 8) and the natural aged combined with treadmill training group (AT group, *n* = 8). The adult rats were used as the control group (YC group, *n* = 8). The body weight of the rats in the YC group was 536.88 ± 30.85, while the body weights of the rats in the AC and AT groups were 649 ± 96.28 and 653.00 ± 33.95 respectively. The experiment used adult and aged rats of matched subline and supplier to control for genetic effects. The rats in the AT group received 8 weeks of treadmill training, and both the AC group and YC group received sham aerobic physical exercise. After 8 weeks of aerobic physical exercise, rats in each group were sacrificed with pentobarbital (50 mg/kg, intraperitoneal) and the tissues were harvested. Each group had eight animals for BMD testing and micro-CT testing. Besides, we randomly chose samples from 6 rats of each group for molecular testing in the present study. The study protocol is shown in Fig. 1.

### Tissue specimen collection

To determine the serum concentration of Procollagen type I carboxy-terminal propeptide (P1CP), C-terminal cross-linking telopeptides of type I collagen (CTX-1), IL-1β, and TNF-α, blood samples collected from the orbit were left standing at room temperature for 2 h and centrifuged (2000 rpm, 20 min) to isolate the serum, which was stored at − 80 °C until use. Right femur and lumbar 5 (L5) vertebrae bones were collected and stored at − 20 °C for bone mineral density (BMD) measurement. The right proximal tibia bones were isolated and stored in a 10% formalin until micro-computed tomography (micro-CT). In addition, the bone marrows of the left femur were extruded by clipping off the epiphyseal ends of the bones and flushing using a needle with PBS (PBS, Gibco), then rapidly frozen with liquid nitrogen for further mRNA and protein analysis [22]. A schematic diagram of tissue collection and detection is shown in Fig. 1B.

### Treadmill training protocol

All the treadmill training was conducted during the day period. During the experimental period, the AT group engaged in corresponding aerobic physical exercise intervention 5 days per week for 8 consecutive weeks. The treadmill training protocol was performed according to the previous reports with mild modification [23, 24]. Prior to training, the aged rats in the AT group were first submitted to the adaptation period consisting of 5 min of treadmill exploration (0 m/minute, 0% incline) followed by 5 min at slow speed (10 m/minute, 0% incline) for the first 3-day of acclimatization. The next day, maximal speed capacity was measured by rising the speed by 5 m/min every 5 min until exhaustion. When the rat’s hindlimbs remained on the electric grid for more than 10 s, they were considered exhausted. During the training stage, the speed was 65% of the maximal speed, which is 12–20 m/min, and the incline was 10% slope. In the first 3 weeks, the rats from AT group started running at a speed of 12–20 m/min and 10% incline for 10 min a day, with exercise duration gradually increasing by 10 min every week. In the second 3 weeks and the third 2 weeks, the training consisted of either 40 min a day or 60 min a day (Fig. 1C). The rats from the YC group and AC groups with sham exercise were placed individually on another treadmill (0 m/s) for the same session number and duration as the AT group. Neither electrical shock nor physical prodding was used to force running in this training stage.

### BMD measurements

As described previously [25], BMDs were measured at the right femur and L5 vertebral body by dual-energy X-ray absorptiometry (DXA) (Lunar, Madison, WI) with software for small animal research.

### Micro-CT analysis

Right tibia (the proximal 2 cm of the tibia) bones were chosen for micro-CT analysis to assess the effect of treadmill training on the maintenance of bone microarchitecture. A 3-mm-thick volume of interest (VOI) was selected 1 mm below the proximal growth plate of the tibia. Micro-CT was performed with a ZKKS micro-CT scanner (Guangzhou Zhongke Kaisheng Medical Technology, Guangzhou, China) according to the manufacturer’s instructions, with a tube voltage of 50 kV, tube current of 0.1 mA, slice thickness of 15 μm, and pixel size of 15 μm. The 3D images were reconstructed using cone beam reconstruction software based on an analysis of a marching cubes-type model with a rendered surface. According to the segmentation and threshold protocol previously described [25], volumetric BMD, trabecular bone volume ratio (BV/TV), bone surface per bone volume (BS/TV), trabecular number (Tb.N), and trabecular separation (Tb.Sp) parameters in the ROI were obtained from the 3D-rendered images of micro-CT for visualization.

### Enzyme-linked immunosorbent assay (ELISA) analysis

ELISA kits were respectively used to measure serum P1CP (CSB-E08081r CSB-E12776r, CUSABIO, Wuhan, China), CTX-1 (CSB-E12776r, CUSABIO), IL-1β(CSB-E08055r, CUSABIO), and TNF-α (CSB-E11987r, CUSABIO).

### Western blot analysis

Tissue samples of the bone marrow of the left femur were dissected and homogenized in RIPA lysis buffer (abiowell, China). Total protein was quantified by a BCA assay (Beyotime), separated by SDS-PAGE and transferred to PVDF membranes (Millipore, USA). The primary antibodies were as follows: rabbit anti-NLRP3 (19771-1-AP, Proteintech, USA), rabbit Anti-proCaspase-1 + p10 + p12 (ab179515, Abcam, UK), rabbit anti-IL-1β (16806-1-AP, Proteintech, USA), rabbit anti-cleaved N-terminal GSDMD(ab215203, Abcam) and mouse anti-β-actin (66009-1-Ig, Proteintech, USA). After rinsing, the membranes were incubated with HRP-conjugated secondary antibodies. The bands on the membranes were developed and fixed by enhanced chemiluminescence (ChemiScope6100, Qinxiang, China) in a dark room. A gel imaging system was used to scan the images and make the film. The bands from the film were finally quantified using Quantity One software (1,709,612, Bio-Rad, USA) and expressed as a ratio to ß-actin (ab8227, Abcam, UK) protein.

### Quantitative real-time PCR

All the procedures were conducted as previously described [25]. Total RNA was extracted from the bone marrow of the left femur using TRIzol (Invitrogen, USA) reagent. Then RNA was reverse-transcribed using a first-strand cDNA synthesis kit (cwbiotech, Beijing, China). Primers for genes including NLRP3, Caspase1, and IL-1β were obtained from singke (Beijing, China). An equal amount of β-actin mRNA was used as an internal standard (singke). The relative expression of each gene was normalized to that of β-actin using the comparative Ct method of quantification (2^−ΔΔCT^ method). The sequences of different primers are listed in Table 1.

**Table 1:** Primer sequences for real-time quantitative PCR

Gene	Forward primer	Reverse primer
β-actin	ACATCCGTAAAGACCTCTATGCC	TACTCCTGCTTGCTGATCCAC
NLRP3	CACCTCTTCTCTGCCTACCTG	AGCTGTAAAATCTCTCGCAGT
Caspase1	CTAGACTACAGATGCCAACCAC	GGCTTCTTATTGGCATGATTCCC
IL-1β	CAGCAGCATCTCGACAAGAG	AAAGAAGGTGCTTGGGTCCT

### Statistical analysis

Data from all experiments were first calculated as mean ± S.E.M. A Kolmogorov–Smirnov test showed that all data were normally distributed. Next, the data were compared by one-way analysis of variance followed by Tukey’s multiple comparisons test. Statistical significance was set at *P* < 0.05, with analyses performed using GraphPad Prism software (version 9.0 c, GraphPad Software, Inc., La Jolla, CA).

## Results

### Treadmill training reduced bone loss in aged rats

**Fig. 2:**
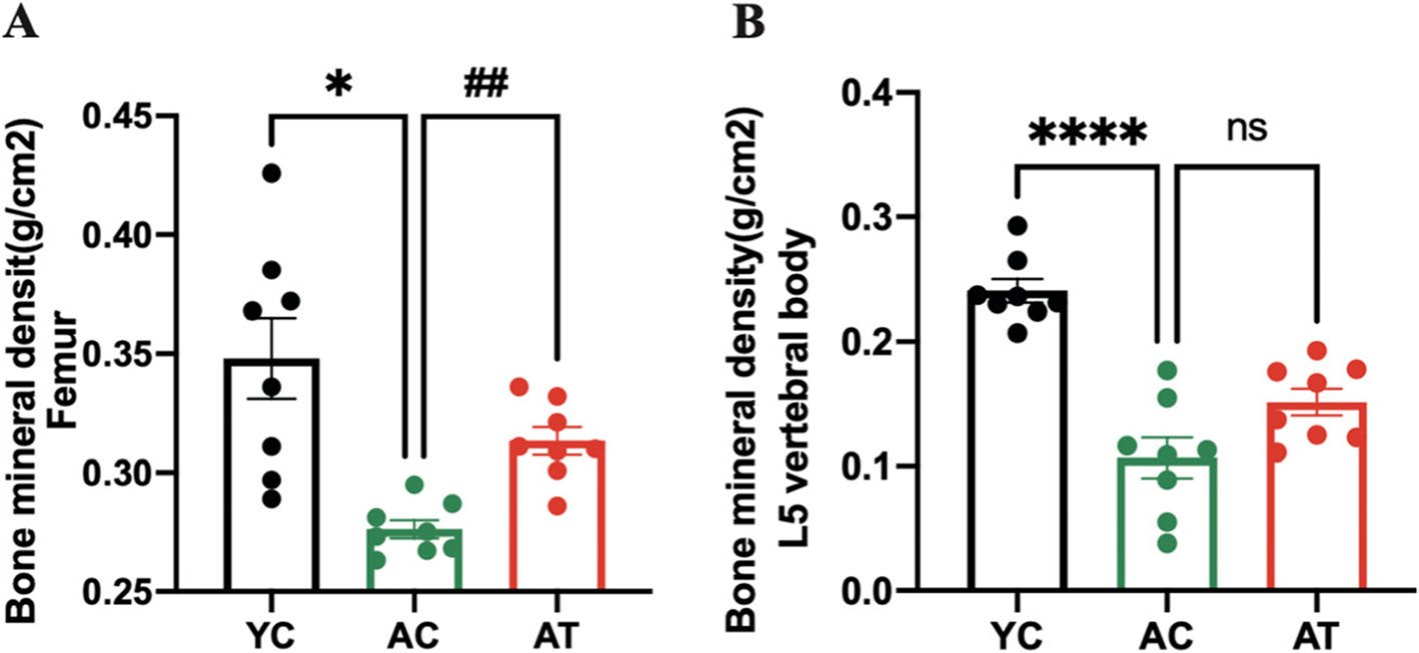
Effects of treadmill training on BMD of the right femur (**A**) and L5 vertebral body **B**. BMD was determined using dual energy X-ray absorptiometry (DXA) after 8 weeks of treadmill training treatment. Data are expressed as mean ± S.E.M (*n* = 8 per group). *P<0.05 and ****P<0.0001 vs. YC group. ##*P*<0.01 vs. AC group. YC, adult rats with sham training; AC, aged rats with sham training; AT, aged rats with real training

We measured the BMD of the right femur and L5 vertebrae body by DXA. As shown in Fig. 2A and B, the aged rats in the AC group had a 1.26-fold and 2.26-fold reduction in BMD of the right femur and L5 vertebrae bone, respectively, in the AC rats than in YC rats (*p* < 0.05, *p* < 0.0001); whereas AT rats had significantly increased BMD by 1.13-fold over AC rats at tibia bone (*p* < 0.01). No significant difference in BMD was observed in L5 vertebrae bone between AT and AC groups (*p* = 0.0848).

### Treadmill training mitigated deterioration of bone microarchitecture in aged rats

**Fig. 3:**
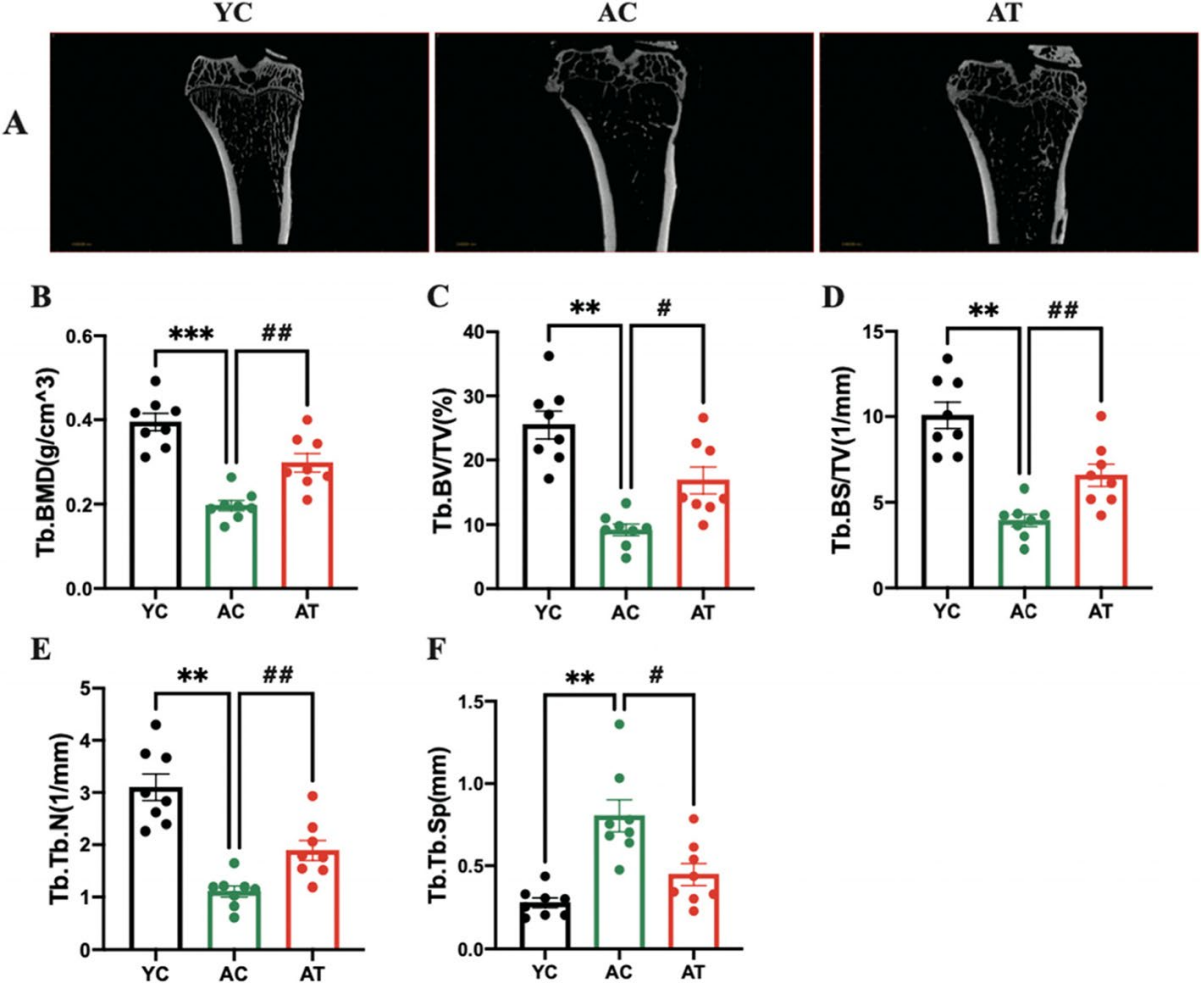
Effects of treadmill training on BMD and micro-CT parameters of the right proximal tibia. **A** Representative images. **B** Trabecular volumetric bone mineral density. Trabecular bone parameters including (**C**) bone volume per tissue volume (BV/TV, %), (**D**) bone surface per bone volume (BS/BV, 1/mm), (**E**) trabecular number (Tb.N, 1/mm), and (**F**) trabecular separation (Tb.Sp, mm) were assessed by quantitative micro-CT. Data are expressed as mean ± S.E.M (*n* = 8 per group). **P<0.01 and ***P<0.001vs. YC group. #*P*<0.05 and ##*P*><0.01 vs. AC group. YC, adult rats with sham training; AC, aged rats with sham training; AT, aged rats with real training

We further analyzed the microstructural properties of the right proximal tibia using micro-CT. Figure 3 illustrates the determination results of bone microstructural parameters of specimens of the proximal tibia obtained from each group. We also measured the volumetric BMD of the right proximal tibia (Fig. 3B) by means of densitometric measurements according to the results of micro-CT, and the results were consistent with the DXA results. We found that BMD of the tibia decreased in the aged rats (*P* < 0.001), and 8 weeks of treadmill training improved the volumetric BMD of the proximal tibia ( *P* < 0.01).

**Fig. 4:**
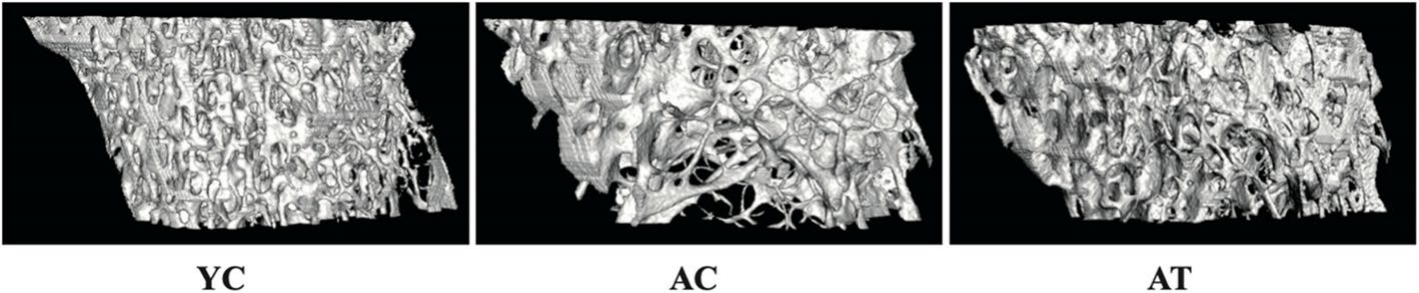
Representative 3D images of the right proximal tibia. YC, AC, and AT, respectively, represent adult rats with sham training, aged rats with sham training, and aged rats with real training

The therapeutic effects on 2-D and 3-D architectural bone changes of the right proximal tibia were respectively shown in Figs. 3A and 4. Analysis of the micro-CT morphometric parameters indicated that the aged rats in the AC group had significantly decreased trabecular BV/TV (2.78-fold, *P* < 0.01), BS/TV (2.55-fold, *P* < 0.01), and Tb.N (2.81-fold, *P* < 0.01) in the proximal tibia respectively, but had a remarkable increase in Tb.Sp (2.90-fold, *P* < 0.01) relative to the YC rats. Eight weeks after treadmill training, AT group exhibited significant differences from AC group in all tibia microarchitectural indices measured by increasing BS/TV (1.84-fold, *P* < 0.05), BS/TV (1.71-fold, *P* < 0.05), Tb.N (1.80-fold, *P* < 0.01), and decreasing Tb.Sp (2.21-fold, *P* < 0.05).

### Treadmill training mitigated ageing-related increase in bone turnover in aged rats

**Fig. 5:**
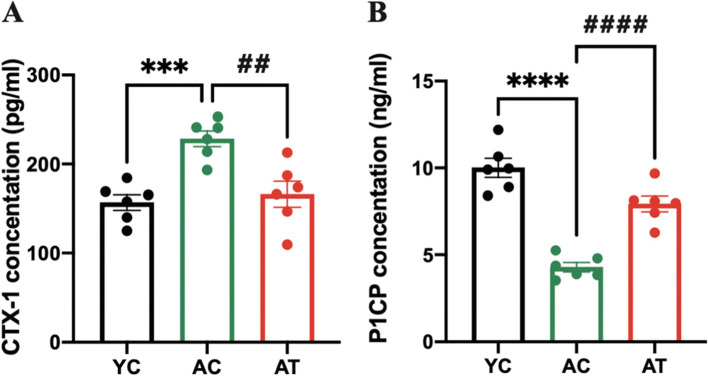
Effects of treadmill training on the serum C-terminal cross-linking telopeptide of type 1 collagen (CTX-1) (**A**) and procollagen type 1 C-terminal propeptide (P1CP) levels (**B**) in aged rats measured by ELISA. Data are expressed as mean ± S.E.M (*n* = 6 per group). ***P<0.01 and ****P<0.0001vs. YC group. ##*P*<0.01 and ####*P*<0.0001 vs. AC group. YC, adult rats with sham training; AC, aged rats with sham training; AT, aged rats with real training

We then analyzed some of the bone metabolic markers involved in bone remodeling to elucidate the mechanism of treadmill training on bone deterioration. Figure 5A and B respectively show the elevated serum osteoresorptive marker CTX-1(1.45-fold, *P* < 0.001) and the falling serum osteogenic marker P1CP (2.33-fold, *P* < 0.0001) in AC rats relative to YC rats, which indicated ageing-related bone turnover is increasing in bone degradation and decreasing in bone formation. After 8 weeks of intervention, AT rats had a 1.85-fold higher serum P1CP level (*P* < 0.0001) and a 1.37-fold lower serum CTX-1 level (*P* < 0.01) as compared to AC rats. The data in Fig. 5 indicates that treadmill training mitigated ageing-related bone turnover by inhibiting bone resorption and promoting bone formation.

### Treadmill training inhibited NLRP3/Caspase1/IL-1β signaling in aged rats

**Fig. 6:**
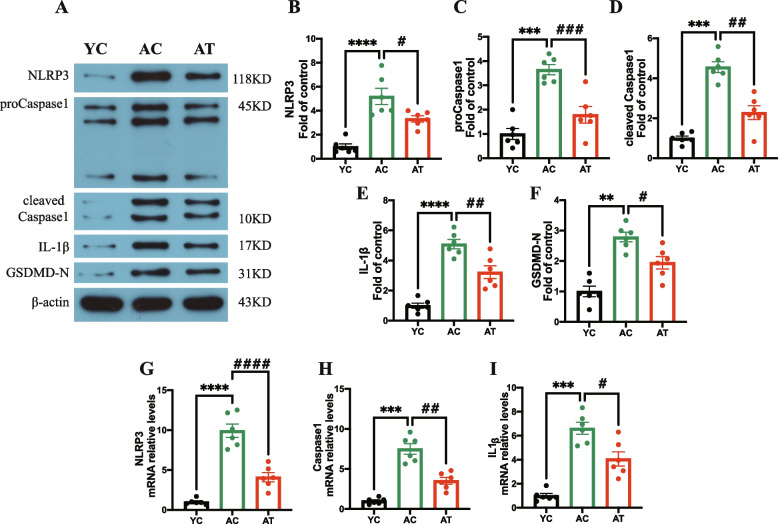
Effects of treadmill training on the NLRP3/Caspase1/IL-1β signaling in bone marrow in aged rats. The cropped gels and blots by western blot of proteins levels of NLRP3, proCaspase1, cleaved Caspase1, IL-1β, GSDMD-N, and β-actin **A**. Quantitative analysis of NLRP3 (**B**), proCaspase1 (**C**), cleaved Caspase1 (**D**), IL-1β (**E**), and GSDMD-N (**F**) in the different groups; The mRNA expression of NLRP3 (**G**), Casepase1 (**H**), and IL-1β (**I**) was detected by qRT-PCR. Data are expressed as mean ± S.E.M (*n* = 6 per group). *P<0.05, **P<0.01, ***P<0.001, and ****P<0.0001 vs. YC group. #*P*<0.05, ##*P*<0.01, ###*P*<0.01, and ####*P*<0.0001 vs. AC group. YC, adult rats with sham training; AC, aged rats with sham training; AT, aged rats with real training. All the samples in this figure are derived from the same experiment and the gels/blots were processed in parallel

Furthermore, we detected the NLRP3/Caspase1/IL-1β signaling in the bone marrow to elucidate the mechanism of treadmill training on ageing-related bone turnover. As shown in Fig. 6A-F, the AC rats showed a dramatic increase by 5.20-, 3.646-, 4.56-, 5.10-, and 2.789- fold respectively in the protein levels of NLRP3, proCaspase1, cleaved Caspase1, IL-1β, and GSDMD-N in bone marrow as compared to YC group (*P* < 0.0001, *P* < 0.001,, *P* < 0.001, *P* < 0.0001, and *P* < 0.01 respectively), Moreover, we found AT group showed a decrease by 1.56-, 2.04-, 2.00-, and 1.58, and 1.43- fold in the protein levels of NLRP3, Caspase1, and IL-1β as compared to AC group (*P* < 0.05, *P* < 0.001, *P* < 0.01, *P* < 0.01, and *P* < 0.05 respectively). These results suggest that treadmill training could inhibit the protein expressions of NLRP3, proCaspase1, cleaved Caspase1, IL-1β, and GSDMD-N.

To validate the above findings, we also analyzed the mRNA levels of NLRP3, Caspase1, and IL-1β in bone marrow as shown in Fig. 6G-I. AC group showed a 9.93-, 7.50- and 6.61-fold increase in mRNA level of NLRP3, Caspase1, and IL-1β as compared to YC group (*P* < 0.0001, *P* < 0.001, *P* < 0.001). After 8 weeks treadmill training, the mRNA levels of NLRP3, Caspase1, and IL-1β in the AT group respectively decreased by 2.42-, 2.12-, and 1.62-fold relative to AC rats (*P* < 0.0001, *P* < 0.01, *P* < 0.05). These results suggest that aerobic physical exercise could inhibit the expression of NLRP3, Caspase1, and IL-1β in bone marrow at the mRNA level. Collectively, the data in Fig. 6 showed that treadmill training could inhibit NLRP3/Caspase1/IL-1β signaling in bone marrow in aged rats.

### Treadmill training alleviated low-grade inflammation in plasma in aged rats

**Fig. 7:**
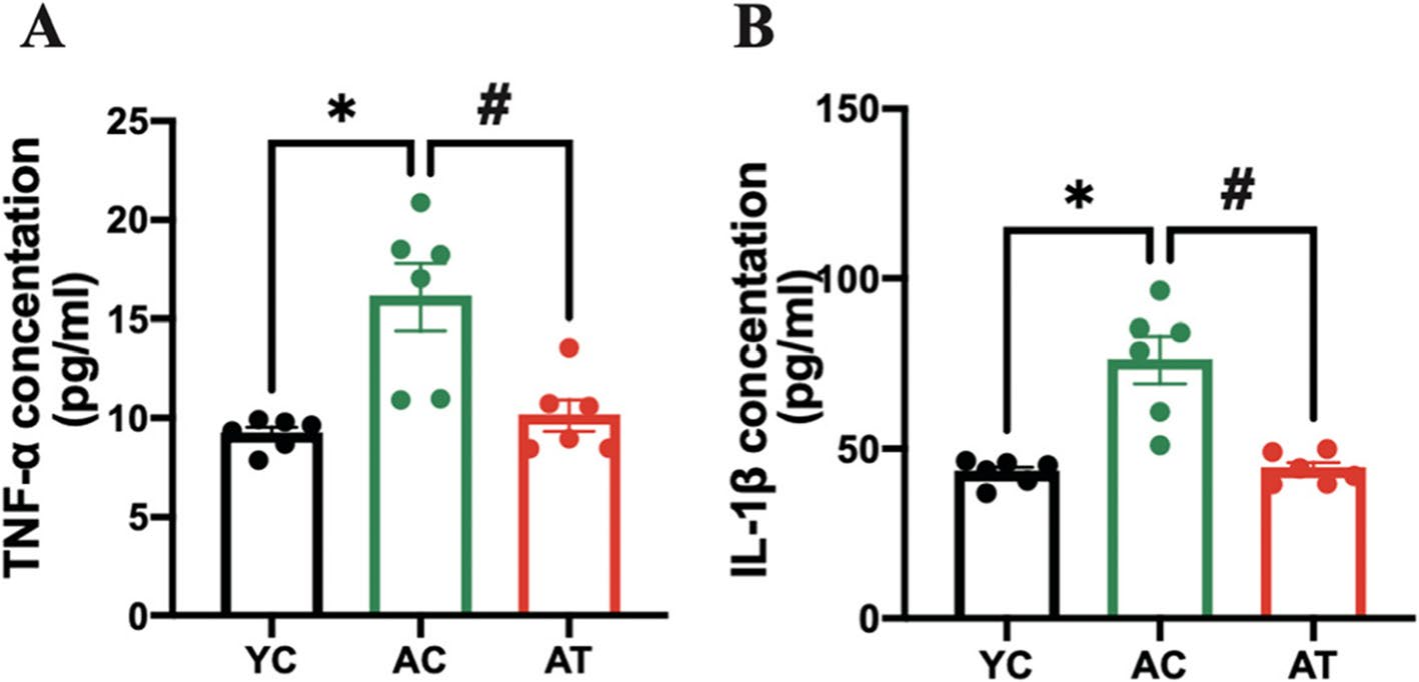
Effects of treadmill training on the serum proinflammatory cytokines in aged rats. The serum concentration of TNF-α (**A**) and IL-1β (**B**) was measured by ELISA. Data are expressed as mean ± S.E.M (*n* = 6 per group). *P<0.05 vs. YC group. #*P<*0.05, vs. AC group. YC, adult rats with sham training; AC, aged rats with sham training; AT, aged rats with real training

We also detect serum proinflammatory cytokines TNF-α and IL-1β, as shown in Fig. 7 to reflect the effect of treadmill training on the inflammatory microenvironment. Compared with the YC group, the AC group showed an increase by 1.71- and 1.75-fold in serum concentrations of TNF-α and IL-1β (*P* < 0.05, *P* < 0.05) respectively. After 8 weeks of treadmill training, serum TNF-α and IL-1β levels in the AT group decreased by 1.75- and 1.72-fold relative to the AC group (*P* < 0.05, *P* < 0.05) respectively. These data indicated that treadmill training could inhibit low-grade inflammation in plasma in aged rats.

## Discussions

**Fig. 8:**
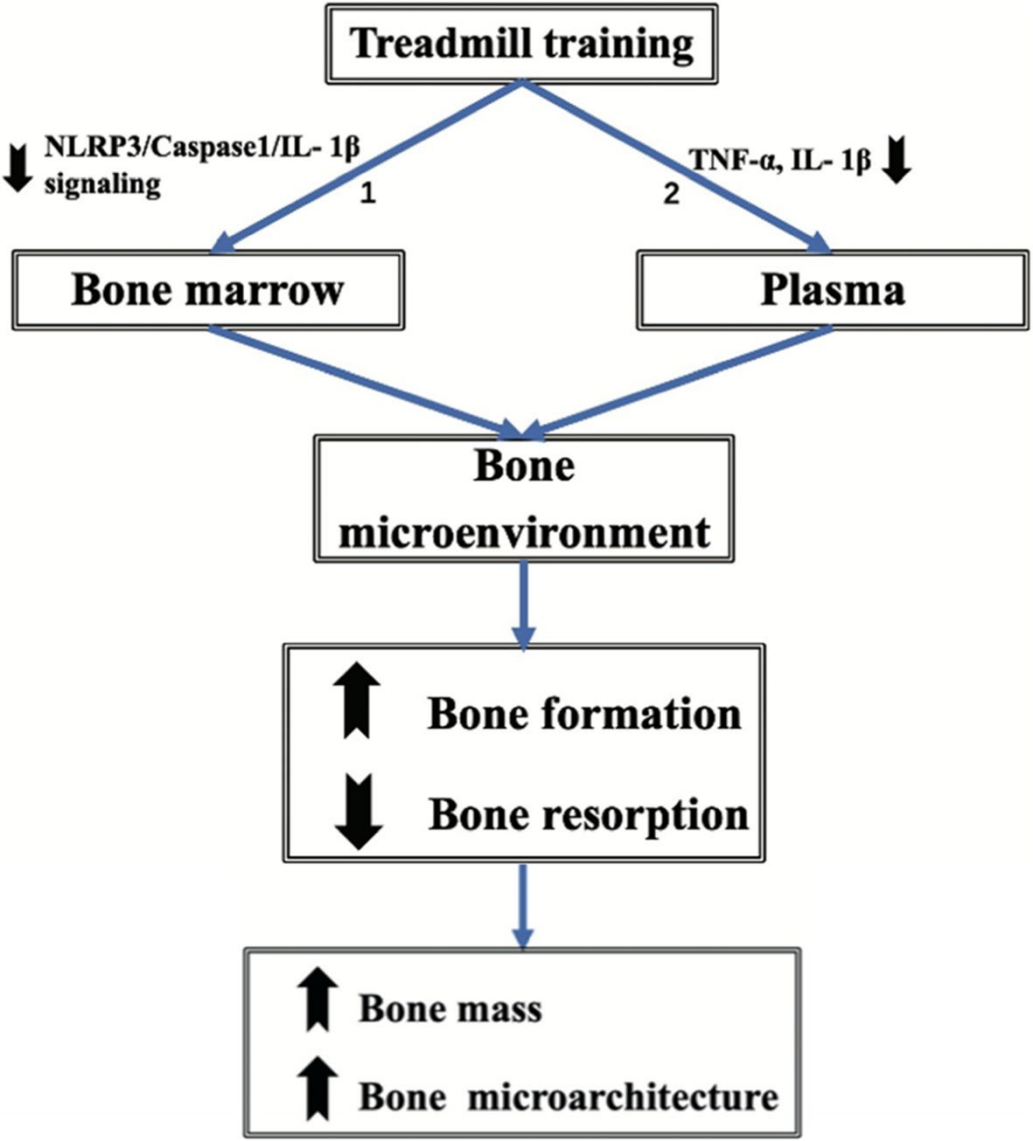
Schematic representation of the action of treadmill training on the bone in aged rats. Treadmill training alters the inflammatory bone microenvironment by inhibiting NLPRP3/Caspase1/IL-1β signaling in bone marrow (1) and downregulating serum TNF-α and IL-1β (2), which in turn improves the bone mass and bone microarchitecture by increasing bone formation and decreasing bone resorption

In the present study, we systematically investigated the effects of treadmill training on ageing-related bone deterioration and NLRP3/Caspase1/IL-1β signaling by detecting changes in bone, bone marrow, and plasma in morphologic, biochemical, and molecular characteristics in a natural ageing rat model, to explore the potential mechanisms by which treadmill training mitigates bone deterioration in aged rats. BMD measurement using DXA is a standard tool in the diagnosis of osteoporosis, used to evaluate the degree of osteoporosis and treatment efficacy. BV/TV, BS/TV, Tb.N and Tb.Sp from micro-CT was used to characterize volumetric BMD and trabecular bone microarchitectural change. Our primary findings are as follows (Fig. 8): 8 weeks of treadmill training improved BMD and bone microarchitecture of hind limb bones (i.e., femur and tibia), accompanied by decreasing bone resorption and increasing bone formation in aged rats. Further investigation showed that treadmill training alleviated inflammatory microenvironment by inhibiting NLRP3, proCaspase1, cleaved Caspase1, IL-1β, and GSDMD-N in bone marrow, as well as TNF-α and IL-1β in plasma. To the best of our knowledge, this is the first study focusing on the effect of mitigating bone loss and alleviating NLRP3/Caspase1/IL-1β signaling of physical activity in ageing rodents.

Factors that contribute to the effect of treadmill training on the ageing skeleton have not been thoroughly explored. Skeletal health is maintained by bone remodeling, a process in which microscopic sites of the effete or damaged bone are degraded on bone surfaces by osteoclasts and subsequently replaced by new bone, which is laid down by osteoblasts [26]. Increasing bone synthesis and decreasing bone degradation by physical activities is considered to be one beneficial factor in the elderly [27]. Biochemical markers in the blood, CTX-1, and P1CP, respectively used to evaluate bone degradation and bone formation, are used to assess the metabolic activity of bone tissue [28, 29]. In this natural ageing rat model, the serum CTX-1 and P1CP ELISA results showed that bone resorption outweighs bone formation with subsequent age-related bone loss in aged rats (Fig. 5A-B). Evidence showed that exercise significantly affected bone metabolism by suppressing CTX-1 and elevating the P1CP level, resulting in an adaptation of bones [30, 31]. We found that 8 weeks of treadmill training partly reversed the increase of serum CTX-1 and decrease of serum P1CP in aged rats accompanied by the improvement of BMD and bone microarchitecture in hind limb bones (Figs. 2, 3 and 4). Therefore, the favorable effect on the improvement of bone turnover by treadmill training is by promoting bone formation and inhibiting ageing-related bone resorption in aged rats. Although serum markers for bone metabolism were investigated in the present study, we did not directly evaluate bone resorption and formation rate. This is a limitation of the study.

In elderly adults or animals, an inflammatory microenvironment induced by a systemic chronic low-grade inflammation, with proinflammatory cytokines increased by 2–4 times, is a common pathophysiological basis for primary osteoporosis [32, 33]. These proinflammatory cytokines have been reported as osteoclastogenic cytokines participating in the activation of the RANKL/RANK/OPG pathway to regulate osteoclasts-mediated bone resorption, thus negatively impacting bone characteristics [34]. Proinflammatory cytokines IL-1β and TNF-α, by inducing expression of RANKL and stimulating RANKL to bind with its receptor RANK, then mediating the proliferation and differentiation of osteoclasts, as well as blocking osteoblast differentiation and osteoblastic bone formation, lead to bone loss in the elderly [35, 36]. In support of this, we measured proinflammatory cytokines in the bone marrow and plasma and found that bone deterioration in aged rats was accompanied by an inflammatory bone microenvironment with an increase in proinflammatory cytokines localized or generalized (Figs. 6 and 7). Evidence has reported that the inflammation-associated bone loss and skeletal stem/progenitor cell decline are reversible [8]. Considering the anti-inflammatory [37] and anti-osteoporosis effects [38] of physical activities in different models, we explore whether the treadmill training had these effects in aged rats. The hypothesis was confirmed by the findings of a profound augmentation in BMD and bone microarchitecture of hind limbs in AT rats (Figs. 2 and 3), and a significant decrease in proinflammatory cytokines in the bone marrow and plasma (Figs. 6 and 7). It seems reasonable that treadmill training has a great beneficial effect on the BMD and bone microarchitecture of hind limbs, at least, partially by inhibiting inflammation both locally and systemically and alleviating the inflammatory bone microenvironment.

Recently, researchers found that the increased damaged macromolecules, organelles, and cell debris can serve as damage-associated molecular patterns (DAMPs) to induce innate immunity through the induction of the canonical NLRP3 inflammasome in ageing, and activated caspase1 processes pro-IL-1β into mature IL-1β, cleaves GSDMD full-length into GSDMD N-terminal, and thereby forming membrane pores to trigger pyroptosis and IL-1β release [39]. NLPRP3/Caspase1/IL-1β pathway regulates age-related bone loss by promoting osteoclastic differentiation [40], and targeted inhibiting NLRP3 reduces age-related bone loss [15]. It is believed that NLRP3/caspase1 pathway plays a critical role in age-dependent inflammatory response [15]. In this study, the higher-level protein of NLRP3, proCaspase1, cleaved Caspase1, IL-1β, and GSDMD-N, and higher-level mRNA of NLPRP3, Caspase1, and IL-1β in the bone marrow of AC rats relative to those of YC rats indicated the NLPRP3/Caspase1/IL-1β pathway-mediated inflammatory bone microenvironment in aged rats, which was consistent with the previous study [40] (Fig. 6). Thus, the maladaptation of the link between NLPRP3/Caspase1/IL-1β signaling-related inflammation and bone turnover may be a key determinant of osteoporosis. Previous studies showed exercise inhibited TLR4/NF-κB/NLRP3 signaling in mice [41]. Therefore, we speculated that inhibition of NLPRP3/Caspase1/IL-1β signaling by exercise may be effective in anti-osteoporosis in aged rats by improving the bone microenvironment. To elucidate the underlying mechanisms responsible for treadmill training for inflammatory bone microenvironment, we measured the NLRP3, proCaspase1, cleaved Caspase1, IL-1β, and GSDMD-N in the bone marrow for aged rats with or without treadmill training. We demonstrated that treadmill training inhibited bone deterioration and inflammatory bone microenvironment, as well as downregulated the expressions of NLRP3, NLRP3, proCaspase1, cleaved Caspase1, IL-1β, and GSDMD-N (Fig. 6). Taken together, these results indicated that the bone deterioration mitigation of treadmill training may be related to the amelioration of inflammatory bone microenvironment by inhibiting NLPRP3/Caspase1/IL-1β signaling in aged rats (Fig. 6), further research is needed in the future to elucidate the potential mechanism.

Importantly, in the current study, treadmill training increased BMD in the femur, but not in the L5 vertebral body (Fig. 2A and B), which we hypothesized may be due to several factors. Firstly, the intensity of aerobic exercise in our study was relatively lower than in the previous studies. Evidence showed that in the aged rat, by 9 weeks, treadmill exercise (treadmill at 17 m/min, 1 h/day, 5 days/week) increased bone mineral content (BMC) and BMD in the tibia, whereas in the vertebrae, only increases in the BMD were found [14]. In addition, 9 weeks of treadmill running (treadmill at 17 m/min, 1 h/day, 5 days/week) could improve the periosteal labeled surface, mineral apposition rate, and bone formation rate in the tibia in aged female rats [42]. Another study demonstrated that 8 weeks of low-intensity treadmill training (treadmill at 12 m/min, 60 min/day, 5 days/week) improved BMD, and it had synergistic effects on BM, structure, and bone strength in ovariectomized, tail-suspended rats [43]. In this study, we adjusted treadmill training by 12–20 m/min and 10% incline, however, the exercise duration was only 10 min in the first week, and no more than 40 min in the second 3 weeks, which is much less than the previous reports. Thus, the insignificant improvement in spine BMD may be due to insufficient training time. In addition, one study showed that 73 days of mild exercise (treadmill at 8 m/min, 1 h/day, 5 days/week) alone did not influence the femur mechanical properties in aged female rats [44], which indicated running speed may be another important parameter for treadmill exercise to mitigate bone deterioration. The condition of the animals, especially the ageing, was the second factor influencing the results of the study. Ageing leads to energy metabolism disorders, such as abnormal glucose metabolism, irregular amino acid metabolism, and aberrant lipid metabolism, exacerbating bone mass loss and inhibiting bone formation [45]. Ageing also leads to a condition, namely sarcopenia, that is characterized by loss of muscle mass, muscle strength, and functional muscle impairment, which aggravates osteoporosis. Besides, sarcopenia-induced decreased mobility and mechanical stress on bone diminish the effect of exercise [46]. Thirdly, Although there was no statistically significant increase in BMD in the L5 vertebral body in the AT group vs. the AC group, it showed an upward trend, which might be due to the limited sample size in this study. Further studies need an adequate sample size. Taken together, treadmill training may help maintain the trabecular bone structure of hind limbs in ageing conditions with certain exercise duration and speed, and additional studies are required to further investigate different exercise parameters and the effects of interaction with the bone microarchitecture.

Although current clinical studies have recommended various exercises to prevent and treat osteoporosis and its complications, the underlying mechanism remains unclear. The chronic inflammatory state can contribute to diseases of ageing such as osteoporosis. In the present study, treadmill training showed mitigation of bone deterioration and suppression of chronic inflammation without adverse effects. Besides, the animal model used here closely approximates the osteoporosis occurrence in the elderly. Our results provide basic evidence to support the future clinical application of treadmill training in the elderly. These findings also extend our understanding of treadmill training, bone deterioration, and inflammatory microenvironment in ageing and suggest possible translation to clinical application in humans.

### Limitation

Several limitations are inherent in the present study. First, our sample size was relatively small, thus the BMD in the L5 vertebral body showed an increasing trend but with no statistical significance. Second, although micro-CT in this study showed an increase in the cortical bone thickness and trabecular bone area, it would be better to show histological analyzes to complement the results. Third, loss of function or inhibition experiments will be done in the future to show that NLRP3 is not merely a correlated finding, but indeed necessary.

## Conclusion

In conclusion, our study offers evidence that treadmill training is an effective intervention to mitigate the ageing skeleton by inhibiting bone loss and reversing the bone microarchitectural deterioration of hind limbs, which is probably by alleviating the inflammatory bone microenvironment regulated by NLPR3/Caspase1/IL-1β signaling. The mitigation of bone deterioration accompanied by inhibition of the NLPR3/Caspase1/IL-1β signaling in the bone marrow and serum proinflammatory cytokines TNF-α and IL-1β suggests a possible molecular mechanism for the effect of treadmill training on the alteration of the inflammatory bone microenvironment in aged rats, which could be considered as a pilot feasibility study needing further experiments. Collectively, treadmill training mitigates the ageing-induced bone loss and reverses the deterioration of bone microarchitecture in hind limbs probably through inhibiting NLRP3/Caspase1/IL-1β signaling to attenuate low-grade inflammation and improve the inflammatory bone microenvironment.

## Supplementary Information

**Additional file 1.**

## Data Availability

All datasets used and/or analyzed during the current study are available from the corresponding author on reasonable request.

## Abbreviations

NLRP3: NOD-like receptor family pyrin domain containing 3
P1CP: Procollagen type I carboxy-terminal propeptide
CTX-1: C-terminal cross-linking telopeptides of type I collagen
BMD: Bone mineral density
BV/TV: Trabecular bone volume ratio
BS/TV: Bone surface per bone volume
Tb.N: Trabecular number
Tb.Sp: and trabecular separation
ELISA: Enzyme-linked immunosorbent assay
IL-1β: Interleukin-1 β
TNF-α: Tumor necrosis factor α
